# Supplementary material for: Live imaging molecular changes in junctional tension upon VE-cadherin in zebrafish
Source: Nat Commun. 2017 Nov 10;8:1402. doi: 10.1038/s41467-017-01325-6 (PMC5680264; doi:10.1038/s41467-017-01325-6)
Supplement: Supplementary file 1 — Supplementary Information [file 41467_2017_1325_MOESM1_ESM.pdf]

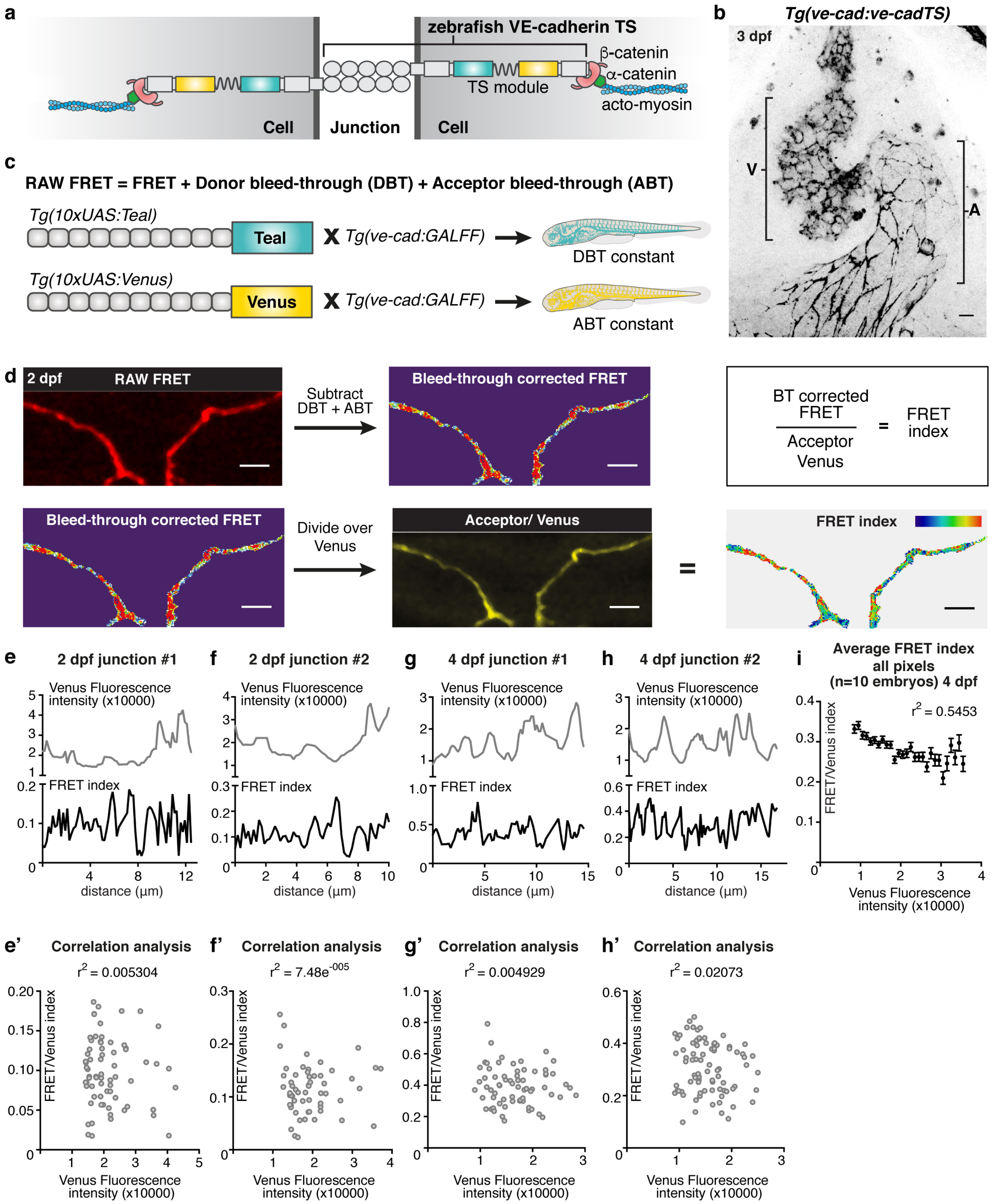
a
zebrafish VE-cadherin TS
β-catenin
α-catenin
TS module
acto-myosin
Cell
Junction
Cell
b
Tg(ve-cad:ve-cadTS)
3 dpf
V
A
c
RAW FRET = FRET + Donor bleed-through (DBT) + Acceptor bleed-through (ABT)
Tg(10xUAS:Teal)
Teal
X Tg(ve-cad:GALFF)
DBT constant
Tg(10xUAS:Venus)
Venus
X Tg(ve-cad:GALFF)
ABT constant
d
2 dpf
RAW FRET
Subtract DBT + ABT
Bleed-through corrected FRET
BT corrected FRET / Acceptor Venus = FRET index
Bleed-through corrected FRET
Divide over Venus
Acceptor/ Venus
FRET index
e
2 dpf junction #1
f
2 dpf junction #2
g
4 dpf junction #1
h
4 dpf junction #2
Venus Fluorescence intensity (x10000)
FRET index
distance (µm)
i
Average FRET index all pixels (n=10 embryos) 4 dpf
r2 = 0.5453
FRET/Venus index
Venus Fluorescence intensity (x10000)
e'
Correlation analysis
r2 = 0.005304
f'
Correlation analysis
r2 = 7.48e-005
g'
Correlation analysis
r2 = 0.004929
h'
Correlation analysis
r2 = 0.02073
FRET/Venus index
Venus Fluorescence intensity (x10000)

Supplementary Figure 1: Controlled FRET quantification of VE-cadherin-TS expression

(**a**) Schematic representation of VE-cadherin Tension Sensor (TS) proteins at a cell-cell junction and intra-cellular linkage to acto-myosin (blue) via β-catenin (red) and α-catenin (green).

(**b**) Venus expression (grey) from *Tg(ve-cad:ve-cadTS)* in the endocardium of the heart at 3 dpf (A=atrial endocardium, V=ventricular endocardium). Scale bar = 10μm.

(**c**) Schematic representation of *Tg(10xUAS:Teal)* and *Tg(10xUAS:Venus)* strains required to correct for Donor bleed through (DBT) and Acceptor bleed through (ABT).

(**d**) Step-wise explanation of RAW FRET processing. From the intensity of each pixel in the RAW FRET image the value for DBT and ABT is subtracted, resulting in the bleed through (BT) corrected FRET. The BT-corrected FRET is subsequently divided by the intensity for Venus in each pixel to normalise for the amount of protein present. The final outcome is represented as a ratio-metric FRET. Scale bar = 5μm.

(**e-f'**) Intensity plots of Venus fluorescence (grey, top) and FRET index (black, below) from spatially matched pixels within a line drawn across single junctions at 2 dpf (**e**-**f**) showing that these quantities to not follow the same profile. Correlation analysis of matched pixels within these junctions is depicted in **e'** (r^2=0.005305, p=0.5491) and **f'** (r^2=7.458^{e-005}, p=0.9487).

(**g-h'**) Intensity plots of Venus fluorescence (grey, top) and FRET index (black, below) from spatially matched pixels within a line drawn across single junctions at 4 dpf (**g**-**h**) showing that these quantities to not follow the same profile. Correlation analysis of matched pixels within these junctions is depicted in **g'** (r^2=0.004929, p=0.5694) and **h'** (r^2=0.02073, p=0.1941).

(**i**) Grouped analysis of ratio-metric FRET values in all pixels within n=52 junctional ROIs selected from n=10 embryos at 4 dpf. Pixels were grouped based on increments of Venus intensity levels of 500. The mean FRET-index value and S.E.M of each Venus intensity group was calculated and plotted. Correlation analysis of mean Venus intensity and mean FRET index within each group (r^2=0.5453, ****p<0.0001).

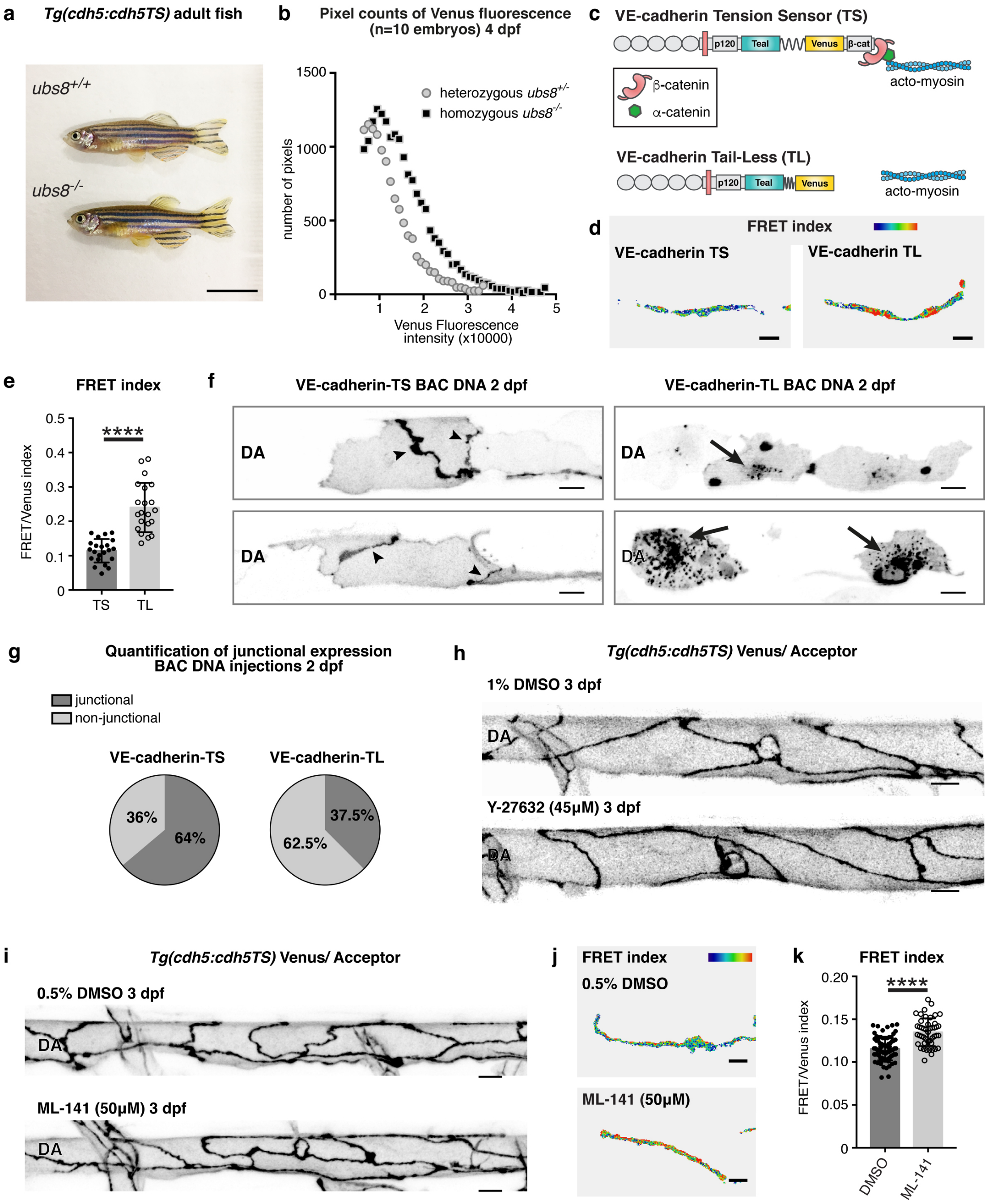

Supplementary Figure 2: The amount of VE-cadherin at the junctions is tightly regulated and under acto-myosin dependent tension

(**a**) Representative image a wild-type (*ubs8*$^{+/+}$) *Tg(ve-cad:ve-cadTS)* animal (top) and a *ve-cadherin* mutant (*ubs8*$^{-/-}$) *Tg(ve-cad:ve-cadTS)* animal showing that mutants are viable and morphologically indistinguishable from genotypic wild-type animals. Scale bar = 1 cm.

(**b**) Quantification of the number of pixels within groups separated by increments of Venus intensity levels of 1000. Each data point is the number of pixels in each group. Pixels numbers are compared between heterozygous *ubs8*$^{+/-}$ (black, pixels in n=47 junctions selected from n=9 embryos) and homozygous *ubs8*$^{-/-}$ (grey, pixels in n=52 junctions selected from n=10 embryos) showing an improved detection range in the homozygous mutants.

(**c**) Schematic representation of VE-cadherin-TS (top) and VE-cadherin-TL protein (bottom) which lacks the β-catenin binding domain (β-catenin=red, α-catenin=green, acto-myosin=blue).

(**d**) Heatmap image of ratio-metric FRET values in junctions expressing VE-cadherin-TS versus VE-cadherin-TL at 2 dpf. Colors range from blue (=low FRET index/high tension) to red (=high FRET index/low tension). Scale bar = 5µm.

(**e**) Ratio-metric FRET values of VE-cadherin-TS (n=22 junctions from n=6 embryos) versus VE-cadherin-TL (n=21 junctions from n=6 embryos) at 2 dpf. Error bars represent mean ± s.d.; ****p<0.0001, from unpaired two-sided t-test.

(**f**) Venus expression (grey) showing representative examples of junctional expression of VE-cadherin-TS (left, black arrowheads) and aggregating VE-cadherin-TL expression (right, black arrowheads) in the DA upon DNA injections. Scale bar = 10µm.

(**g**) Quantification of junctional versus non-junctional expression in cells expressing VE-cadherin-TS (n=72 cells analysed) and VE-cadherin-TL (n=32 cells analysed) at 2 dpf.

(**h**) Junctional morphology of ECs in the DA (Venus, grey) at 3 dpf in 1%DMSO and Y-27632 (45µM/1%DMSO) treated embryos. Scale bar = 10µm.

(**i**) Junctional morphology of ECs in DA (Venus, grey) at 3 dpf in 0.5%DMSO and ML-141 (50µM/0.5%DMSO, Cdc42 inhibitor) treated embryos. Scale bar = 10µm.

(**j**) Heatmap image of ratio-metric FRET values in junctions of 0.5%DMSO and ML-141 (50µM/0.5%DMSO) treated embryos at 3 dpf. Colors range from blue (=low FRET index/high tension) to red (=high FRET index/low tension). Scale bar = 5µm.

(**k**) Ratio-metric FRET values in junctions from 0.5%DMSO treated controls (n=81 junctional ROIs from n=9 embryos) and ML-141 (50µM/0.5%DMSO) treated embryos (n=46 junctional ROIs from n=5 embryos) at 3 dpf. Error bars represent mean ± s.d.; DMSO – ML-141 ****$p<0.0001$, from unpaired two-sided t-test.

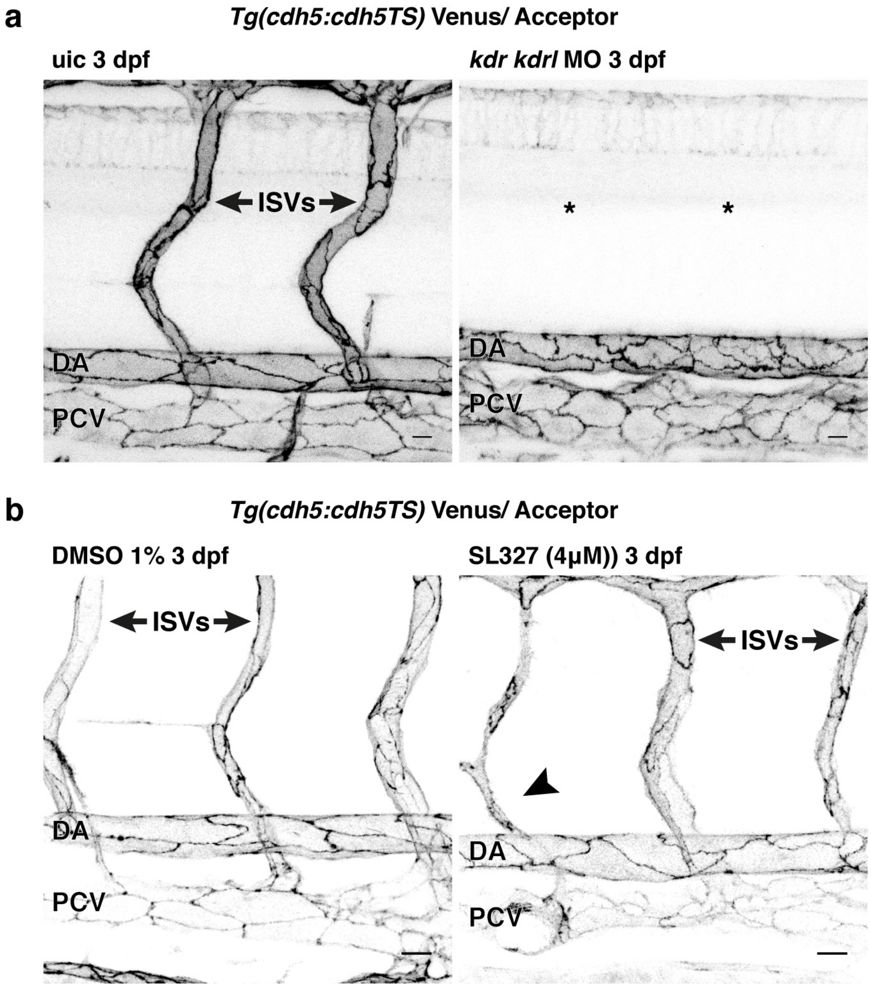

Supplementary Figure 3: Gross phenotypes in *kdr/kdrl* morphant and SL327 treated embryos

(**a**) Venus expression (grey) from *Tg(ve-cad:ve-cadTS)* in the trunk of an un-injected control (uic) and a *kdr/kdrl* MO injected embryo at 3 dpf showing a loss of intersegmental vessels (ISVs) in the morphant (asterisks). Scale bar = 10µm.

(**b**) Venus expression (grey) at 3 dpf from *Tg(ve-cad:ve-cadTS)* in 1%DMSO treated control and SL327 (4µM/1%DMSO) treated embryos. Treatment from 20 hpf till 3 dpf. SL327 treatment results in only mild intersegmental vessel defects (compared to *kdr/kdrl* knockdown (**a**)) recognized by thin ISVs (black arrowhead). Scale bar = 10µm.

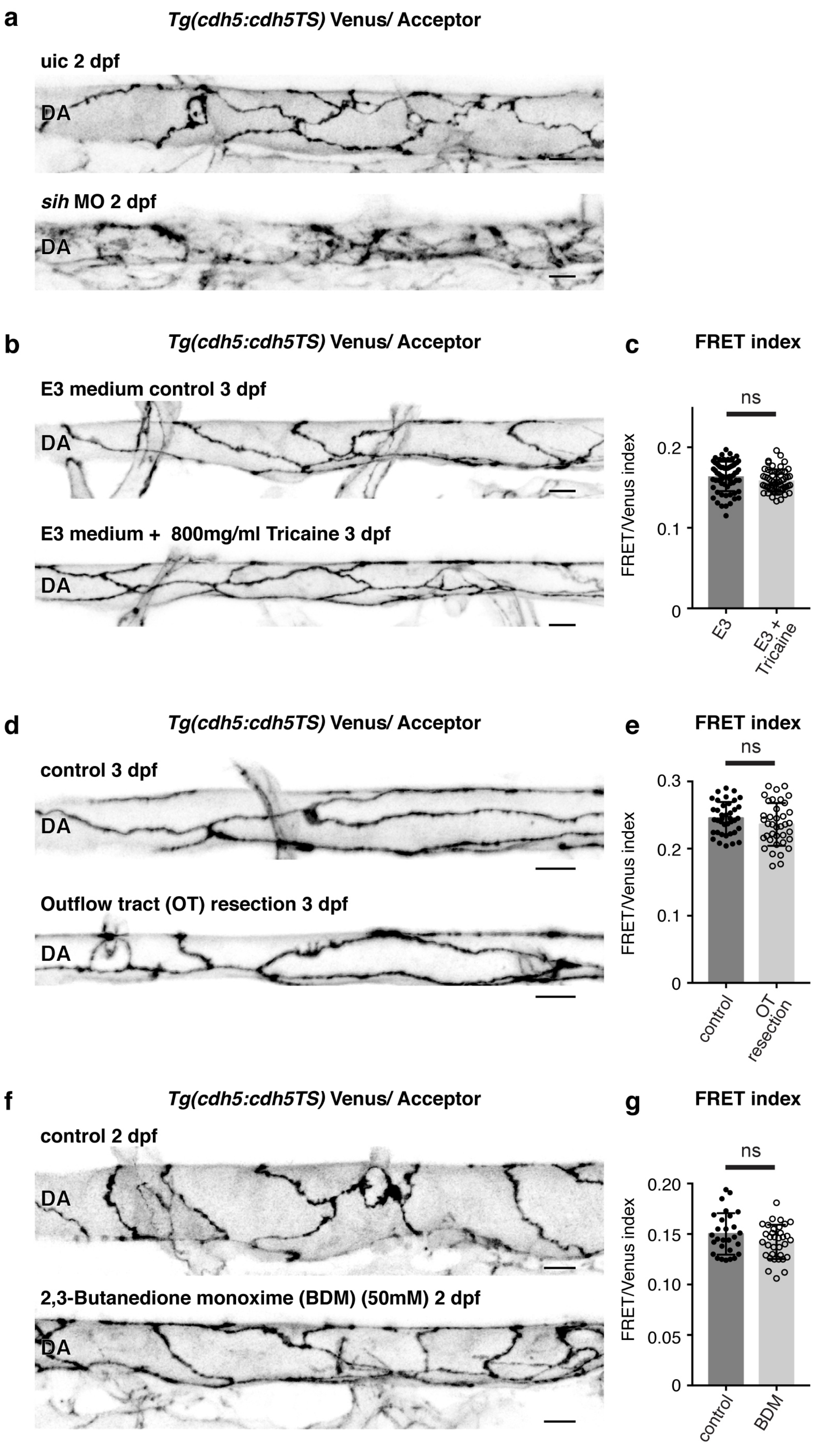
a
Tg(cdh5:cdh5TS) Venus/ Acceptor
uic 2 dpf
DA
sih MO 2 dpf
DA
b
Tg(cdh5:cdh5TS) Venus/ Acceptor
E3 medium control 3 dpf
DA
E3 medium + 800mg/ml Tricaine 3 dpf
DA
c
FRET index
ns
FRET/Venus index
0.2
0.1
0
E3
E3 + Tricaine
d
Tg(cdh5:cdh5TS) Venus/ Acceptor
control 3 dpf
DA
Outflow tract (OT) resection 3 dpf
DA
e
FRET index
ns
FRET/Venus index
0.3
0.2
0.1
0
control
OT resection
f
Tg(cdh5:cdh5TS) Venus/ Acceptor
control 2 dpf
DA
2,3-Butanedione monoxime (BDM) (50mM) 2 dpf
DA
g
FRET index
ns
FRET/Venus index
0.20
0.15
0.10
0.05
0
control
BDM

Supplementary Figure 4: Acute loss of blood flow does not inflict immediate changes in tension across VE-cadherin

(**a**) Junctional morphology of ECs in the DA (Venus, grey) of an uic and a *sih* MO injected embryo at 2 dpf showing that mature junctions fail to form without flow. Scale bar = 10µm.

(**b**) Morphology of junctions in the DA (Venus, grey) of a control embryo and an embryo that has been incubated in a high dose of Tricaine (800mg/ml) to stop cardiac contraction and blood flow. Scale bar = 10µm.

(**c**) Ratio-metric FRET values in junctions of control (n=68 junctional ROIs from n=10 embryos) versus Tricaine treated embryos control (n=56 junctional ROIs from n=10 embryos). Error bars represent mean ± s.d.; not significant (ns) p=0.0834, from unpaired two-sided t-test.

(**d**) Venus expression (grey) showing junctional morphology of ECs in the DA at 3 dpf of a control embryo and an embryo that underwent surgical outflow tract resection to stop blood flow. Image taken 3 hours after surgery. Scale bar = 10µm.

(**e**) Ratio-metric FRET quantifications from control embryos (n=39 junctional ROIs from n=8 embryos) and embryos in which the outflow tract was surgically disconnected (n=38 junctional ROIs from n=7 embryos). Error bars represent mean ± s.d.; not significant (ns) p=0.01472, from unpaired two-sided t-test.

(**f**) Morphology of junctions in the DA (Venus, grey) of an untreated control versus a 2,3-butanedione monoxime (BDM, 50mM) treated embryo at 2 dpf. Scale bar = 10µm.

(**g**) Ratio-metric FRET quantifications from junctions in untreated embryos (n=28 junctional ROIs from n=8 embryos) versus junctions in BDM (50mM) treated embryos (n=34 junctional ROIs from n=8 embryos) at 2 dpf. Error bars represent mean ± s.d.; not significant (ns) p=0.1039, from unpaired two-sided t-test.
