# Supplementary material for: Live imaging molecular changes in junctional tension upon VE-cadherin in zebrafish
Source: Nat Commun. 2017 Nov 10;8:1402. doi: 10.1038/s41467-017-01325-6 (PMC5680264; doi:10.1038/s41467-017-01325-6)
Supplement: Supplementary file 3 — Description of Additional Supplementary Files [file 41467_2017_1325_MOESM3_ESM.pdf]

Description of Additional Supplementary Files

File Name: Supplementary Movie 1
Description: **Representative video of *kdr/kdrl* morphants with intact blood circulation.** Left: Video of bright field z-stack taken from un-injected control embryo at 3 dpf showing circulating blood cells. Right: Representative video of bright field z-stack taken from *kdr*/*kdrl* MO injected embryo at 3 dpf showing blood circulation.
